# Cochlear SGN neurons elevate pain thresholds in response to music

**DOI:** 10.1038/s41598-021-93969-0

**Published:** 2021-07-15

**Authors:** R. I. M. Dunbar, Eiluned Pearce, Bronwyn Tarr, Adarsh Makdani, Joshua Bamford, Sharon Smith, Francis McGlone

**Affiliations:** 1 Department of Experimental Psychology, University of Oxford, Anna Watts Building, Radcliffe Observatory Quarter, Oxford, OX1 6GG UK; 2 Division of Psychiatry, University College London, London, WC1A 4AA UK; 3 Institute of Cognitive and Evolutionary Anthropology, University of Oxford, Banbury Road, Oxford, OX2 6PE UK; 4 Research Centre Brain and Behaviour, Liverpool John Moores University, Liverpool, L3 3AF UK; 5 Institute of Psychology, Health and Society, University of Liverpool, Liverpool, UK

**Keywords:** Neuroscience, Auditory system, Cochlea

## Abstract

The C-tactile (CLTM) peripheral nervous system is involved in social bonding in primates and humans through its capacity to trigger the brain’s endorphin system. Since the mammalian cochlea has an unusually high density of similar neurons (type-II spiral ganglion neurons, SGNs), we hypothesise that their function may have been exploited for social bonding by co-opting head movements in response to music and other rhythmic movements of the head in social contexts. Music provides one of many cultural behavioural mechanisms for ‘virtual grooming’ in that it is used to trigger the endorphin system with many people simultaneously so as to bond both dyadic relationships and large groups. Changes in pain threshold across an activity are a convenient proxy assay for endorphin uptake in the brain, and we use this, in two experiments, to show that pain thresholds are higher when nodding the head than when sitting still.

## Introduction

Afferent low-threshold c-fibre mechanoreceptors (CLTM) are a class of highly specialised unmyelinated peripheral nerves that, when stimulated by a low force, low velocity ‘caressing’ stimulus delivered at skin temperature, induce a state of positive affect^1–3^. Microneurography recordings from CLTMs in humans find that their firing frequency, in response to brushing touch, mirrors psychophysical ratings of touch “pleasantness”^4^. CLTM afferents have been found only in hairy skin and project to the posterior insular cortex rather than to the primary somatosensory (SI) cortex that is the primary target of myelinated touch nerves^5,6^. These properties support a role for CLTMs in regulating homeostatic positive affective states. Gentle dynamic touch to the skin of the body increases endogenous opioidergic activity in the brain^7^ and may also release oxytocin^8^.

Although the roles of endorphins and oxytocin have not so far been adequately dissociated in experimental studies^9^, it is clear that this kind of dynamic touch induces a state of wellbeing by stimulating dopamine in the nucleus accumbens, increases social interactions, reduces anxiety by action on the amygdala, decreases stress by action on the hypothalamic–pituitary–adrenal axis (HPA-axis)^10^, and reduces pain in term infants undergoing a heel-lance^11^.

In primates, social grooming is the central behavioural mechanism that creates social bonding^12^. The sweeping hand movements used in grooming are the preferred stimulus to activate the receptors of cutaneous CLTM neurons^1,4,13^, and neurobiological experiments confirm that these movements trigger an endorphin response in the brain in both primates^14–17^ and humans^7,18^, playing a central role in driving the reward associated with close physical contact, thereby enhancing social bonding^19–22^. A recent rodent study reported that daily stroking, specifically targeted to activate CLTMs, also confers resilience against established markers of chronic unpredictable mild stress^10^. In a study that measured the effects of tactile and kinaesthetic stimulation in premature infants, Field et al.^23^ reported significant positive effects on a range of developmental measures.

Recent anatomical studies have identified a high density of unmyelinated type-II spiral-ganglion (SGN) neurons in the mammalian cochlea and the Organ of Corti that share a genetic signature with these C-type afferent neurons^24,25^. Since, unlike the type-I SGNs, they appear to be insensitive to sound, the function of these neurons remains unclear, although it has been suggested that they could have an effect similar to peripheral CT fibres^24,26^. Other functions have, however, been suggested. Noting that these type-II neurons are mainly confined to the basal half of the cochlea, where they would be most sensitive to low frequency sounds, Wu et al.^24^ suggested that one function might be to buffer the organism against painful sounds in this range, although this seems incompatible with the role these neurons play elsewhere in the hairy skin^27^.

Given how frequently rhythmic head movements occur in human social interactions (e.g. during laughter, when expressing assent and encouragement, while listening to or engaging with music) as well as the frequency of rocking in autistic and orphanage children, an alternative possibility is that they play a key role in triggering the endorphin system in the context of social interactions, especially in the context of music-induced movement. Indeed, head movements, in particular, may be integral for perceiving music’s rhythmic structure^27,28^. Such movements might plausibly trigger the endorphin system via these cochlear receptors, and so might account for the activation of the endorphin system while listening to (as opposed to performing) music and, through that, for our enjoyment of music. Full body motion during dancing is known to raise pain thresholds and enhance social bonding^29,30^, with the involvement of the endorphin system in this confirmed experimentally by administration of naltrexone (an endorphin antagonist)^18^. This provides a *prima facie* case for suggesting that head movements alone might enable this if endorphin activation is triggered by the cochlear SGNs. Some evidence for this suggestion is provided by the fact that rocking induces sleep in rodents^31^ as well as triggering a vestibular response that has been shown to calm human neonates^32^: in both cases, the only substantive movement involves the head.

That pain thresholds, endorphin activation and enhanced bonding are causally related has been demonstrated experimentally for several other explicitly social activities (e.g. laughter, with endorphin uptake confirmed by PET neuroimaging^7^). A plausible hypothesis, then, is that any functionally related receptors in the cochlea might subserve a similar function, either as a primary function or as a derivative function of some other primary function.

In two studies, we test whether head movement elevates pain thresholds in the same way as has been shown to be the case by full-body movement during dancing^17,29,30^. In this, we follow the widely employed validated practice of using changes in pain threshold as a proxy for endorphin activation in the brain^33,34^.

## Experiment 1

This experiment aimed to determine whether nodding the head in time to music while holding the rest of the body still was sufficient to trigger an endorphin response in the brain, as indexed by a change in pain threshold.

### Methods

Forty one subjects (63.4% females; mean age = 25.0 ± 8.1 years, range 18–62: 88% European or North American) were asked either to nod their head naturally in time to music (experimental condition) or to sit completely still listening to the same music (control condition). The music was a 13-min compilation from a range of up-beat contemporary dance music and was of a type that would naturally invite spontaneous movement. The length of the clip was determined by the fact that previous studies with other equivalent activities (laughter, singing, dancing) suggest that at least 10 min action is required to ensure a response in terms of pain threshold.

Subjects were seated comfortably at a desktop computer and listened to the music through earphones. They were asked not to move any part of the body, other than their head in the experimental condition. The (spoken) instructions given to the subjects can be found in the Online SI.

To ensure that subjects followed the instructions about head nodding, their head movements were recorded using an accelerometer. In the experimental condition, six subjects failed to nod their head sufficiently vigorously to lift the vertical trace above baseline, and in the control condition two subjects moved their heads significantly despite being asked not to do so (for examples of correct and incorrect responses by subjects in the experimental condition, see *SI*). Given that our concern is with whether subjects nod their heads rather than whether they listen to music per se, we analysed the data two ways: first, by condition, with the eight subjects who failed to adhere to the instructions excluded (N = 33), and, second, since all subjects listened to the same music, partitioning all 41 subjects by whether or not they nodded their head sufficiently vigorously to lift the vertical trace above baseline.

Before and after taking part in the experiment, subjects had their pain threshold assessed using the Roman chair (or wall-sit) task that has previously been used in many similar experiments^29,30,35,36^. The duration subjects held the position was timed on a stopwatch. All experiments that have used this or similar pain threshold tasks as an assay have found that pain threshold always declines in the control condition (i.e. in the absence of any intervention), such that the change in threshold is usually significantly negative. The appropriate baseline for comparison, then, is the negative change in the control condition, not zero change. Since the hypothesis is explicitly directional, 1-tailed statistical tests are appropriate.

Before starting the experiment, subjects were asked to rate on a sliding scale (0–100) how much they listened to music in the average day, how often they played a musical instrument or sang in a choir, and how musical they considered themselves to be. After completing the experiment, they answered three questions relating to their enjoyment of the music they had just heard: how familiar was the music, how much they enjoyed it and how similar the music was to their preferred music.

All statistical analyses were executed in SPSS v.25.

The data are provided in the ESM-Dataset. The stimulus files are available on request.

Ethical approval was granted by the Combined University Research Ethics Committee of the University of Oxford. All subjects provided informed consent; all methods were performed in accordance with the relevant guidelines and regulations.

### Results and discussion

**Figure 1:**
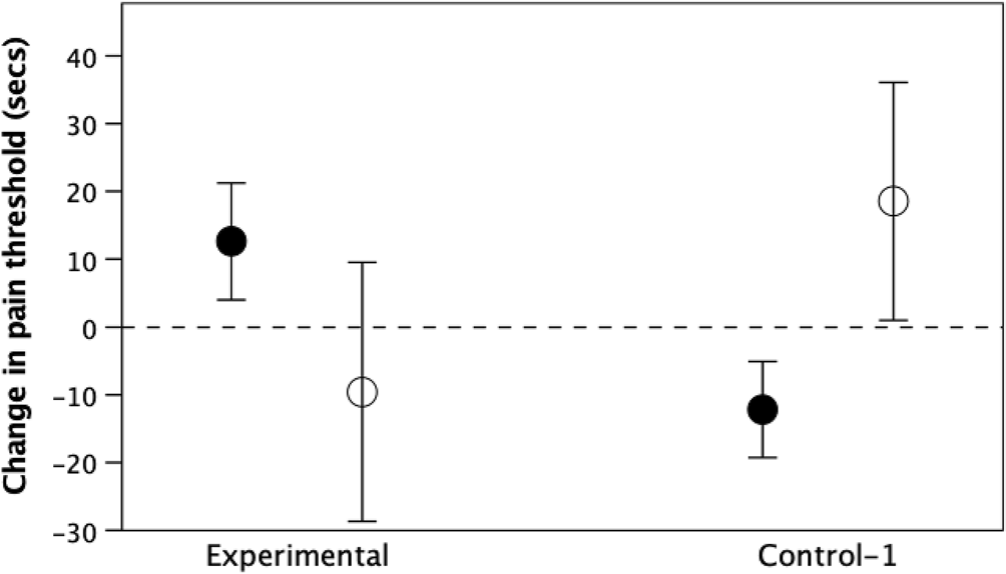
Mean (± 1 se) change in absolute pain threshold following the music task. Filled symbols: subjects who complied with the instructions they were given (nodding their head in the experimental condition, remaining still in the control condition); unfilled symbols: subjects who failed to follow instructions (failed to head-nod in the experimental condition or head-nodded in the control condition). Sample size (L to R): 14, 6, 19, 2.

Figure 1 plots the absolute change in pain threshold under the two conditions, with subjects differentiated according to whether they followed the instructions correctly (solid symbols) or did not follow the instructions (i.e. nodded their head when they were asked to remain still, or failed to nod when asked to do so: unfilled symbols). For those subjects who followed the instructions correctly, the mean difference from before to after in the duration for which the position was held in the experimental condition was + 12.64 ± 32.1SD s (against a mean pre-task duration of 63.1 ± 22.1 s), compared to − 12.2 ± 31.0SD s (against a mean pre-task duration of 81.8 ± 54.6 s) in the control condition. Despite the small sample size, the difference between the two conditions is significant (F_1,31_ = 5.01, p = 0.033 1-tailed): subjects who rhythmically nodded their head in time to the music (experimental condition) had a larger change in pain threshold than those who remained still (control-1 condition).

Figure 1 also shows (as unfilled symbols) the data for subjects that failed to follow instructions (i.e. failed to nod in the experimental condition, or nodded in the control condition when they had been instructed not to do so). Note how closely these erroneous performances match those in the opposite condition. Partitioning the data simply by whether or not vigorous head nodding occurred increases the significance of the difference (F_1,39_ = 6.57, p = 0.014).

As a check to ensure that the sample size was not too small to yield reliable statistical results, we ran a post hoc power analysis and determined effect sizes for each condition (Table 1). Power > 0.8 is generally considered acceptable. Effect size for the experimental condition is also high. We are less concerned about the lower power on the control condition since we do not expect the change in pain tolerance to differ significantly from 0. The results thus seem robust despite the small sample.

**Table 1:** Post-hoc power analyses for Figs. 1 and 2 for p = 0.05 (1-tailed).

Experiment	Condition	Power	N	Effect size (η^2^)
1	Experimental-1	0.846*	14	0.752
1	Control-2^‡^	0.051^†^	19	0.021
2	Experimental-1	0.976^†^	35	0.625
2	Control-3	0.937^†^	35	0.548
2	Control-4	0.881^†^	39	0.461

*Against mean observed change in control-2 condition.

^†^Against 0 change.

^‡^Two-tailed.

There were no differences between the two conditions in how much subjects listened to music (t_31_ = 1.38, p = 0.176), how often they played instruments or sang in choirs (t_31_ = 0.73, p = 0.469), or how musical they rated themselves to be (t_31_ = 0.26, p = 0.796). Nor were there any differences between the two conditions in the subjects’ post-task ratings of the music’s familiarity (t_31_ = 0.365, p = 0.519), their enjoyment of the music (t_31_ = 1.22, p = 0.231), or their preference for the particular kind of music heard (t_31_ = − 0.83, p = 0.413).

Overall, the scores on the three musical experience variables did not correlate significantly with pain threshold (Spearman correlations: r = − 0.14, p = 0.454; r = − 0.24, p = 0.178, r = − 0.25, p = 0.168, respectively). Nor did pain threshold change correlate with the post-task ratings of how familiar the music was (r = − 0.081, p = 0.655), how much they enjoyed the music (r = 0.01, p = 0.941) or how similar the music was to their preferred musical tastes (r = 0.22, p = 0.213).

Taken together, these results confirm the hypothesis that head nodding in time to music raises pain thresholds, indicating that the endorphin system had been activated. Note that the negative change in the control condition is just on the margin of being significantly < 0 (directional one sample *t* test, t_18[∂duration=0]_ = − 1.71, p = 0.052). This negative change in pain threshold in the control condition mirrors, and is of very similar magnitude to, that observed in the control conditions of many other previous experiments that have assayed pain threshold change using different ways of measuring pain (cold pressor task, sphygmomanometer, Roman chair) and a variety of stimulus tasks (including laughter^37^, singing^21,35^, dancing^29,30^, storytelling^36^ and the rituals of religion^38,39^).

These results cannot be explained by differences in musicality, or by individual differences in how familiar the music was or how much the subjects enjoyed the music, since these do not differ significantly between conditions. Recent research has reported that attention has been found to attenuate perception of pain^40^, and our results might be due to this effect if the two conditions differed in attention. This seems an unlikely explanation for two reasons: (1) subjects are likely to have had to concentrate harder in the control condition in order to resist the natural tendency to move the body rhythmically in response to music, and this ought t have elevated pain thresholds rather than reduce them, and (2) the pain stimuli were experienced after attending to the stimuli rather than whilst attending to them.

The one outstanding question, however, is whether the music itself plays a causal role in elevating pain thresholds or the effect is due simply to the physical activity. This was tested in the follow-up experiment.

## Experiment 2

Experiment 2 compared two new control conditions with the same experimental condition as Experiment 1. One control condition used head-nodding to nature sounds rather than music (control-2), and the other used foot-tapping to the same music rather than head-nodding (control-3). It’s aim was to determine whether any relevant physical movement triggers an endorphin response or whether the music itself plays a seminal role.

There were four possible outcomes of interest:

1. The two control conditions have significantly less effect on pain threshold than the experimental condition: this would imply a seminal role for music in some way (although this might simply relate to its role in providing a rhythm for nodding);
2. Head nodding has a significantly greater effect on pain threshold than toe-tapping, irrespective of whether it is done to music or not: this would imply that the music itself was irrelevant, with nodding the head being more effective than rhythmic actions using another part of the body (in this case, toe-tapping);
3. Head nodding and toe-tapping to music had a significantly greater effect on pain threshold than head nodding to non-musical arrhythmic sounds: the music is important (perhaps because it helps maintain a rhythm) but which part of the body is moved may not be important;
4. All three result in equally elevated pain thresholds: this would imply that head nodding in any form (rhythmic or arrhythmic) will elevate pain thresholds, and is neither more nor less effective than rhythmic movement of any other part of the body.

### Methods

For this experiment, 119 subjects (66.4% females, mean age = 25.0 ± 6.7 years, range 18–49; 82% European or North American) were asked either to nod their head in time to the music (*experimental* condition, as in Experiment 1), to nod their head in time to random, arrhythmic nature sounds (*control-2*) or to tap one foot in time to the same music as in the experimental condition (*control-3*). Since our concern is to determine whether the experimental condition (head-nodding to music) differs from other forms of physical movement to sound, we did not include the stationary (“no nodding”) control condition: we know from Experiment-1 and many other previous experiments^36,37^ that this condition has a negative effect on the duration for which the Roman Chair position is held. For each condition, we compare observed change in pain threshold against the mean observed change (− 12.2 s) in the control-1 condition in Experiment-1. Subjects were videoed to ensure that they carried out the required action correctly.

Eight subjects were excluded either because they declined to undertake the pain threshold task or because they arrived at the experimental venue immediately after sustained physical exercise or because they failed to head-nod at all (in the *control-2* condition only, thanks mainly to lack of the rhythm in the sound).

All statistical analyses were executed in SPSS v.25.

The data are provided in the *ESM-Dataset.*

Ethical approval was granted by the Combined University Research Ethics Committee of the University of Oxford. All subjects provided informed consent; all methods were performed in accordance with the relevant guidelines and regulations.

### Results and discussion

**Figure 2:**
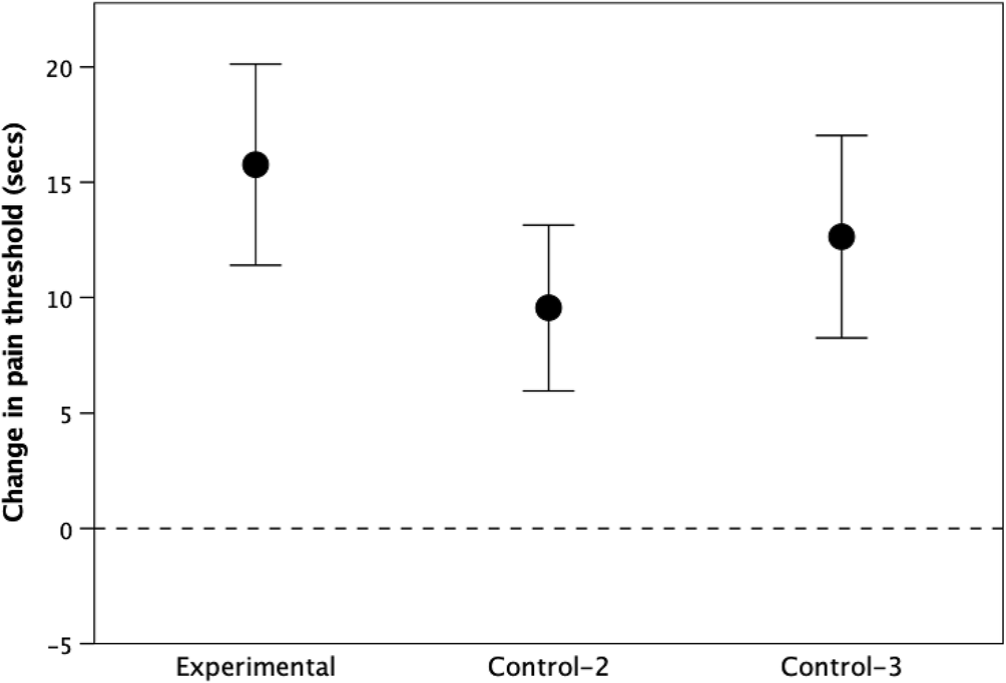
Mean (± 1 se) change in pain threshold following nodding in time to the same music as used in Experiment 1 (Experimental), nodding to arrhythmic nature sounds (control-2) or tapping a foot to the same music as in the experimental condition (control-3). Sample sizes are: 36, 32, 39 (L to R).

Figure 2 plots the change in pain threshold (normalised to the mean change of − 12.2 s in the control condition in Experiment 1) in the three conditions. In all three cases, pain threshold increased significantly following the activity (directional one sample *t* tests against H_0_ = − 12.2 s: experimental, t_32_ = 3.71, p = 0.0005; control-3, t_32_ = 3.26, p = 0.006; control-4, t_8_ = 2.88, p = 0.004). The change was also significantly > 0 in all three cases (p < 0.007). Although both control groups scored slightly lower than the experimental condition, the differences between the three conditions were not significant (F_2,106_ = 0.29, p = 0.749). Post hoc power analyses and effect sizes are given in Table 1, and are acceptably high.

As in Experiment 1, there were no significant differences between the experimental and the two control groups in how much music subjects listened to music (F_2,106_ = 1.19, p = 0.307), how often they played or sang (F_2,106_ = 1.58, p = 0.212), or how musical they rated themselves to be (F_2,106_ = 0.41, p = 0.664), thus ruling these variables out as possible confounds. Not too surprisingly, there were significant differences between the conditions in the post-task ratings of enjoyment (F_2,106_ = 3.11, p = 0.049) and the preference for the kind of music they heard (F_2,106_ = 4.04, p = 0.020), but, not surprisingly, this was entirely due to those who listened to random nature sounds. There were no differences between the three groups in the music’s familiarity (F_2,106_ = 1.83, p = 0.166).

As before, there were no correlations between change in pain threshold and any of the three musical experience questions (everyday listening to music: r = − 0.07, N = 109, p = 0.490; playing music: r = − 0.06, p = 0.816; self-rated musicality: r = − 0.15, p = 0.123). Nor did pain threshold change correlate with the post-task ratings of how familiar the music was (r = − 0.002, p = 0.983), how much subjects enjoyed the music (r = − 0.16, p = 0.092) or how similar the music was to their preferred music (r = − 0.02, p = 0.810).

These results suggest that the music does not itself contribute directly to the elevated pain thresholds, since nodding to arrhythmic non-music also raised pain thresholds (albeit to a lower extent than when rhythmically nodding to music). Moreover, it seems that any kind of rhythmic action (in this case, foot-tapping) will also elevate pain thresholds, in this case presumably via the CT neuron system that permeates the skin. These results thus support option (4) above.

## General discussion

Taken together, the two experiments confirm that movement of the head activates the endorphin system sufficiently to elevate pain thresholds in a manner similar to that reported for a number of other social activities (including laughter, singing and dancing^18,21,29,30^). This provides *prima facie* evidence that one function of the cochlea SGNs may be to trigger the central endorphin system. These results complement findings that rocking induces sleep in rodents^31^ as well as human neonates^32^, a sleepy state being one outcome of endorphin uptake in the brain. These effects also parallel findings that light stroking reduces responses to stress in rodents^10^ and the perception of pain in human babies^11^.

Experiment 2 suggests that the music on its own only has a weak direct effect over head-nodding to non-music and foot-tapping to music. In other words, it seems to be the physical movement of any body part that generates the endorphin up-regulation. However, music might still play an important indirect role by providing a basis for maintaining both rhythmic consistency and synchrony between individuals when ‘musicking’ together. This might also allow an individual to maintain rhythmic activity for longer than they might otherwise be motivated to do. Although the mechanisms involved are not well understood, behavioural synchrony turns out to have a very significant effect on endorphin up-regulation: synchrony in physical activities such as rowing has been shown to elevate pain thresholds significantly above that caused by the physical activity on its own^41^, and the same is true of dancing^29,30^. The release of endorphins might explain why music is both so rewarding and so effective in bonding communities^35,42^. The fact that SGNs are most dense in the apical part of the cochlea may explain why bass instruments are so commonly used to provide the rhythmic accompaniment to music.

SGNs in the inner ear appear to be a mammal-wide phenomenon and absent in birds^25^. This would suggest that their origin lies in some common feature of mammalian biology. It is difficult to see what that might be. Although buffering the auditory system against painful sound in the low frequency range has been suggested^24^, this seems unlikely given the enormous range of variation in the basal frequency of hearing across the mouse-to-elephant curve. An alternative, perhaps more plausible, suggestion might have to do with the repetitive head movements that all mammal mothers make when licking and nuzzling their newborn infants—and often continue to make while nuzzling their infants after weaning. Since the endorphin system is known to be involved in mother-infant bonding in mammals^43–48^ and to reduce distress in primate and human infants^10,11^, endorphins triggered by head movements might help to reinforce the bonds between mother and infant. Importantly, endorphins have a much longer half-life in the central nervous system than oxytocin (21 h^49^ vs 1.9–6.3 min^50,51^, respectively), and this is likely to have important consequences for the persistence of any behavioural or cognitive effects^52^.

The sensitivity to gentle touch is even present prenatally with foetuses responding to maternal touch on the mother’s abdomen^53^. An intriguing hypothesis by Bystrova^54^ proposes that rhythmic stimulation of lanugo hairs during foetal rocking movement by the amniotic fluid stimulates CLTM nerves, inducing a priming effect on the developing social brain via neuropeptide release. By extension, and of relevance for this report, if these in-utero tactile experiences engage reward systems in the foetus, then vestibular sensation associated with movement in the womb may serve as a secondary reinforcer, contributing to the comfort neonates subsequently derive from rocking, bouncing, and swaying—movements with a similar frequency to the preferred stimulus for CLTMs. In support of a putative function of vestibular stimulation, it is well known that maternal carrying of distressed infants while moving reduces crying and heart rate over and above the (also significant) effect of static maternal holding alone. In this context, it is worth noting that, in whole body rocking of infants, the infant is usually held tightly such that other parts of the body do not move in a way that deforms the skin or the CT receptors; as a result, any response to rocking will be mainly experienced by the inner ear mechanisms as the head is moved through space.

Either way, it seems that, in humans at least, this system has been co-opted during recent evolution to underpin the social bonding role of music (a cultural phenomenon)^20,29^, conceivably via an intermediate step in which maternal singing (or humming) functioned to calm infants^55,56^. It is not until they are about 12 months postpartum that human infants reach the same developmental stage achieved at birth by the infants of monkeys and apes (who are not rocked by their mothers)^57^, and it is during this period that human infants are most in need of soothing. If so, we might infer that the exaptation of the cochlea mechanism for a role in music can only date from the point at which hominins evolved the modern human reproductive pattern. This appears to be around 500,000 years ago with the appearance of archaic humans^58^.

Finally, these findings may go some way to explaining stereotypical behaviours such as rocking that occur in autistic spectrum individuals^59,60^ as well as in caged animals^61^. Such behaviour is associated with reduced stress and elevated endorphin levels^62,63^. The findings reported herein suggest that truncal rocking movements may well trigger the endorphin system via the cochlea SGNs, providing a calming response to stressful situations.

## Supplementary Information

Supplementary Information 1.

Supplementary Information 2.
