# Author Correction: Cochlear SGN neurons elevate pain thresholds in response to music

**DOI:** 10.1038/s41598-022-04978-6

**Published:** 2022-01-11

**Authors:** R. I. M. Dunbar, Eiluned Pearce, Bronwyn Tarr, Adarsh Makdani, Joshua Bamford, Sharon Smith, Francis McGlone

**Affiliations:** 1 Department of Experimental Psychology, University of Oxford, Anna Watts Building, Radcliffe Observatory Quarter, Oxford, OX1 6GG UK; 2 Division of Psychiatry, University College London, London, WC1A 4AA UK; 3 Institute of Cognitive and Evolutionary Anthropology, University of Oxford, Banbury Road, Oxford, OX2 6PE UK; 4 Research Centre Brain and Behaviour, Liverpool John Moores University, Liverpool, L3 3AF UK; 5 Institute of Psychology, Health and Society, University of Liverpool, Liverpool, UK

Correction to: *Scientific Reports* https://doi.org/10.1038/s41598-021-93969-0, published online 15 July 2021

The original version of this Article contained an error.

In the ‘General discussion’ section,

“The fact that SGNs are most dense in the lower part of the cochlea may explain why bass instruments are so commonly used to provide the rhythmic accompaniment to music.”

now reads:

“The fact that SGNs are most dense in the apical part of the cochlea may explain why bass instruments are so commonly used to provide the rhythmic accompaniment to music.”

The original Article has been corrected.

