# Supplementary material for: Cochlear SGN neurons elevate pain thresholds in response to music
Source: Sci Rep. 2021 Jul 15;11:14547. doi: 10.1038/s41598-021-93969-0 (PMC8282857; doi:10.1038/s41598-021-93969-0)
Supplement: Supplementary file 1 — Supplementary Information 1. [file 41598_2021_93969_MOESM1_ESM.docx]

**Cochlear SGN Neurons Elevate Pain Thresholds in Response to Music**

**R.I.M. Dunbar, Eiluned Pearce, Bronwyn Tarr, Adarsh Makdani, Joshua Bamford, Sharon Smith & Francis McGlone**

***Supplementary Information***

**Spoken instructions given to subjects before starting the music task**

*You are now going to do a simple task whilst listening to an audio clip which will last about 10 minutes.

To begin, let's make sure you are sitting in a relaxed position.

If your legs are crossed, please uncross them now and place your feet flat on the floor. Your knees should be bent about 90 degrees, and your feet should be pointing forwards. Please take this position now, and make sure you are comfortable. Try and maintain this posture throughout the task.

Next, place your hands in your lap, and close your eyes.*

*Once the audio clip begins, you will do a simple task. It’s important that you follow these instructions carefully…*

| **Condition** | **Instructions immediately before the audio clip** | **Clip**  (9 mins) |
| --- | --- | --- |
| Head movement music | *Maintaining the relaxed posture, please nod your head up and down while listening to the audio clip. Try not to move any other parts of your body, except your head. While listening to the audio clip, nod your head, as feels natural.* | Music |
| No movement control  [Control-1] | *Maintaining the relaxed posture, please listen to the audio clip. Try not to move your body. While listening to the audio clip, maintain a natural still position.* | Music |
| Head movement non-music control  [Control-2] | *Maintaining the relaxed posture, please nod your head up and down while listening to the audio clip. Try not to move any other parts of your body, except your head. While listening to the audio clip, nod your head, as feels natural.* | Nature sounds |
| Non-head movement music control  [Control-3] | *Maintaining the relaxed posture, please tap your toes up and down while listening to the audio clip. Try not to move any other parts of your body, except your feet. While listening to the audio clip, tap your toes, as feels natural.* | Music |

**Compliance with instructions to head nod**

Examples of the accelerometer traces for two typical subjects in the Experimental condition in Experimenta-1, one of whom correctly followed instructions to nod and the other who failed to nod, is shown below. The Y-axis deflection (shown in orange) represents vertical deflection of the head.

**Head nodding in response to music**


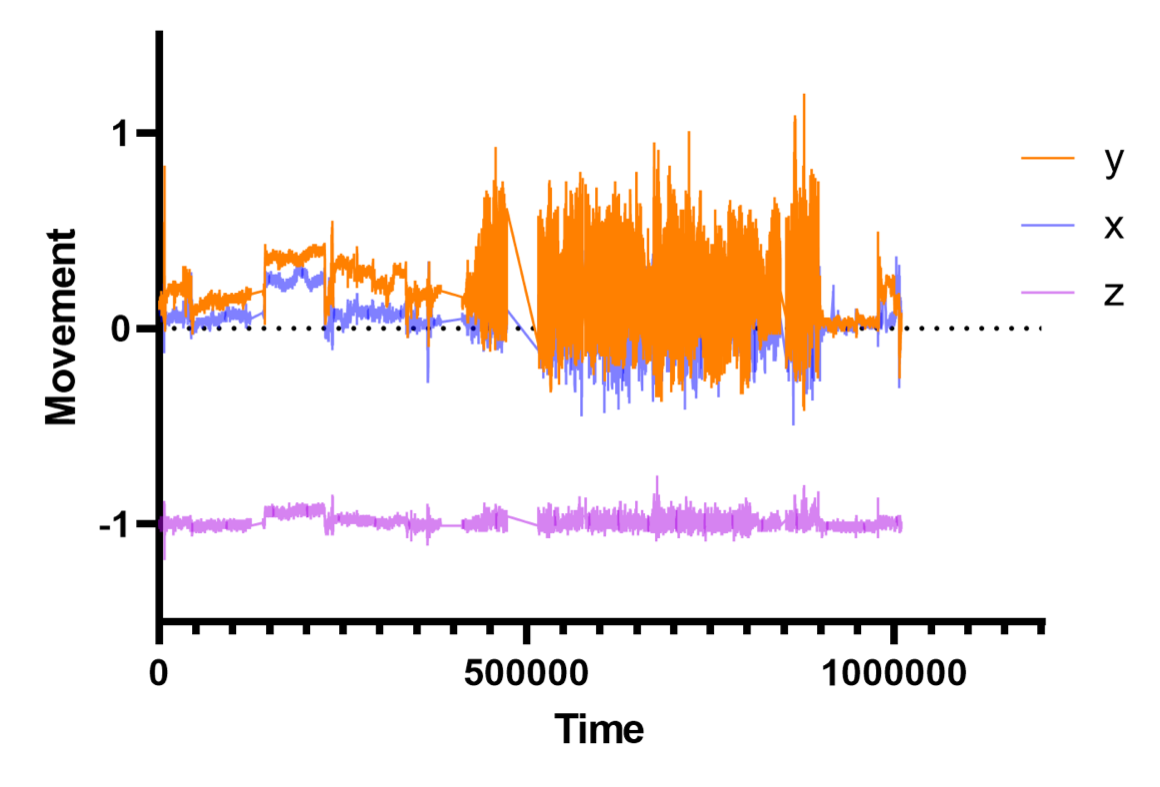


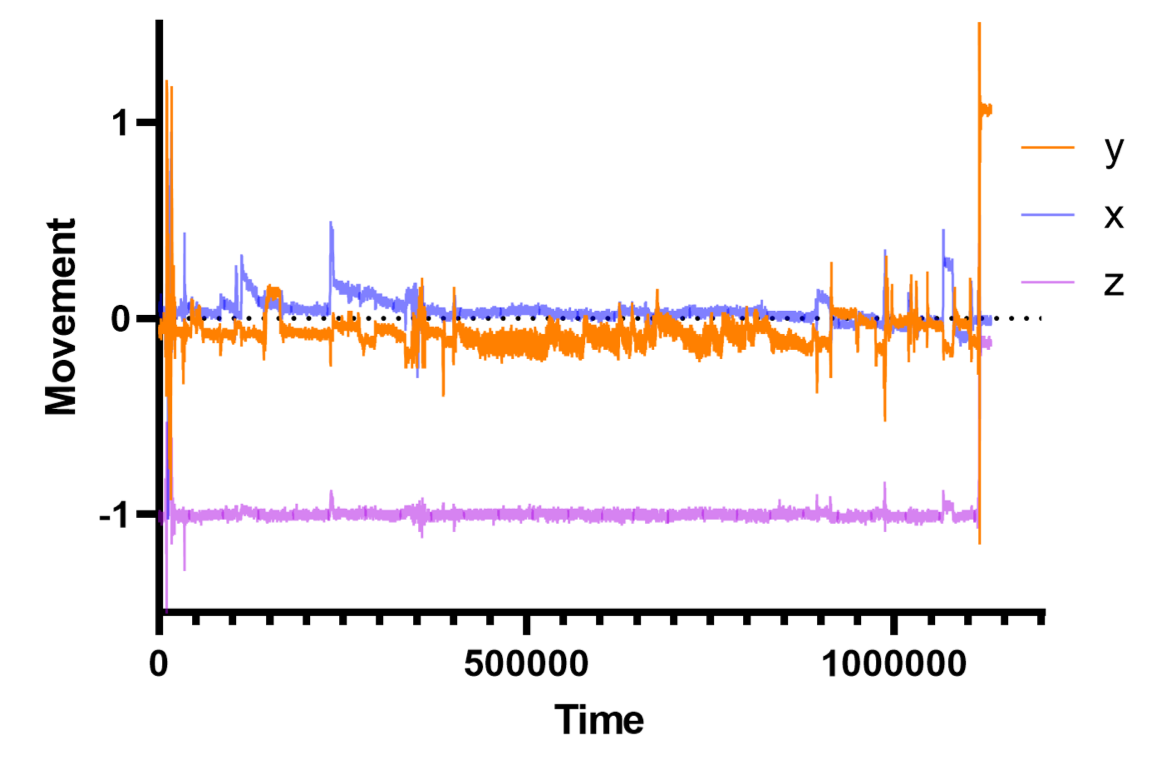


**Failure to head nod**
